# The Association of Hematological and Biochemical Parameters With Mortality Among COVID-19 Patients: A Retrospective Study From North India

**DOI:** 10.7759/cureus.29198

**Published:** 2022-09-15

**Authors:** Asha K S., Vandini Singh, Yatiraj Singi, Rajesh Ranjan

**Affiliations:** 1 Department of Biochemistry, All India Institute of Medical Sciences, Raipur, Raipur, IND; 2 Department of Physiology, Maharishi Markandeshwar Institute of Medical Sciences and Research, Ambala, IND; 3 Department of Forensic Medicine and Toxicology, All India Institute of Medical Sciences, Bilaspur, Bilaspur, IND; 4 Department of Community Medicine, Noida International Institute of Medical Sciences, Gautam Buddh Nagar, IND

**Keywords:** hematological parameters, disease outcome, clinical feature, retrospective, covid-19

## Abstract

Background

There have been reports published in the Indian setting that describe demographics, clinical characteristics, hospital course, morbidity, and death in coronavirus disease 2019 (COVID-19) patients; however, they are based on small numbers of cases. The current analysis of patients with known outcomes allowed us to gain a better understanding of the disease process and progression in COVID-19 patients, as well as correlate the factors that influence the outcome.

Methods

This was a record-based, retrospective observational study of patients admitted to a COVID-19 hospital (All India Institute of Medical Sciences (AIIMS), Raipur, India). Between June 1 and August 31, 2021, we gathered medical records of all hospitalized patients having a laboratory-confirmed COVID-19 diagnosis and a known outcome (discharged or died). The extracted data included basic demographics, signs and symptoms, duration of hospitalization, laboratory parameters, and outcomes. Categorical variables were analyzed using either the chi‑square test or Fisher’s exact test. The level of significance was set at p<0.05.

Results

The mean age of the patients was 53.77±15.85 years. Of the patients, 84.2% have moderate to severe disease, and 15.8% of the patients have mild disease. Furthermore, 26.3% of the subjects were deceased, while 73.7% were discharged. The laboratory parameters that were significantly (p<0.05) raised among the dead compared to discharged patients included serum total bilirubin (mg/dL), serum direct bilirubin (mg/dL), serum indirect bilirubin (mg/dL), serum urea (mg/dL), serum uric acid (mg/dL), hematocrit (%), total leukocyte counts (/mm^3^), neutrophils (%), serum sodium (Na) (mEq/L), serum chloride (Cl) (mEq/L), and phosphate (mg/L).

Conclusion

Clinical and laboratory features reflect disease pathophysiology and hence assist doctors in determining the severity of medical sickness. They also help in the creation of clinical care management algorithms that may improve patient outcomes.

## Introduction

Coronavirus disease 2019 (COVID-19) is a viral sickness that mostly affects the respiratory tract. The two preceding coronavirus outbreaks were in 2003 with the severe acute respiratory syndrome-related coronavirus (SARS-CoV) infection and in 2012 with the Middle East respiratory coronavirus (MERS-CoV) epidemic [1]. In March 2020, the World Health Organization (WHO) labeled this disease a pandemic due to its global spread.

Fever, cough, dyspnea, and infiltrations in the chest are the most common symptoms of COVID-19 infection [2,3]. Despite the fact that the majority of infections discovered are not hazardous, 15%-20% of COVID-19 patients may develop a life-threatening illness, such as respiratory arrest, shock, or multiple organ failure, requiring hospitalization to an intensive care unit [4,5].

Severe acute respiratory infection (SARI) is defined by the World Health Organization as a respiratory illness with a fever or body temperature of 38°C or above, coughs, and onset within the last 10 days that warrants hospitalization [6]. All SARI patients should be checked for COVID-19 with a high index of suspicion in the midst of the current pandemic. Using all COVID-19 patients as a denominator allows researchers to examine the clinical pattern in mild COVID-19 patients and moderate to severe COVID-19 patients.

The Indian Council of Medical Research (ICMR) published recommendations in March 2020 to investigate all patients with SARI for COVID-19 [7].

Numerous hospitals throughout the country have been recognized as COVID-19 facilities to handle confirmed or suspected COVID-19 patients. When the disease reached pandemic proportions in India, our hospital (All India Institute of Medical Sciences (AIIMS), Raipur, India) was designated as an official location for managing COVID-19 patients. More than 400 beds were made dedicatedly available for COVID-19 confirmed or suspected cases. The confirmation of COVID-19 cases was done using reverse transcription-polymerase chain reaction (RT-PCR) assays performed in the microbiology department.

Given the disease’s fast expansion and rising number of patients, the disease’s full clinical course remains unknown for Indian patients. There have been reports published in the Indian setting addressing demography, clinical features, inpatient outcome, complication, and survival in patients; however, they are based on small numbers of cases [8-10]. The current analysis of patients with known outcomes allowed us to gain a better understanding of the disease process and prognosis in COVID-19 patients, as well as to correlate the factors that determine the outcome. This will aid in triaging the rapidly increasing number of patients and simplifying resources for improved case management and improved performance in future COVID-19 waves.

## Materials and methods

Study design and population

This was a record-based, retrospective observational study of patients admitted to AIIMS, Raipur, India (more than 400 beds), which was designated as a COVID-19 hospital by the State Government. Over 1,500 patients were admitted to the hospital until the first week of August 2021. Between June 1 and August 31, 2021, we collected digital and paper-based patient records for all hospital admissions with RT-PCR-confirmed COVID-19-positive patients and a known outcome (discharged or died).

The inclusion criteria were as follows: positive results on real-time reverse transcription-polymerase chain reaction (RT-PCR) assay of nasal and pharyngeal swab specimens for severe acute respiratory syndrome coronavirus 2 (SARS-CoV-2), age > 18 years (male and nonpregnant, nonlactating female), and closed cases with known outcome.

The government’s testing guidelines were followed. Many patients with a confirmed COVID-19 disease were unable to isolate at residence and had been taken to the hospital there at the time of this study. This research examined severe, moderate, and mild diseased patients. All RT-PCR-positive patients were subdivided into two groups, one with an oxygen saturation of >94% (mild group) and the other with an oxygen saturation of <94% (moderate-to-severe group).

Data collection

Basic demographic characteristics (age and gender) were extracted, as well as signs and symptoms (cough, fever, breathlessness, anosmia, headache, myalgia, pneumonia features (such as chest pain, difficulty breathing, wheezing, chest in drawing, and history of tuberculosis (TB)), gastroenteritis features (such as nausea, vomiting, abdominal discomfort, and diarrhea), ear fullness or pain, dizziness, and convulsions), comorbidities (diabetes, hypertension, asthma, chronic obstructive pulmonary disease (COPD), chronic kidney disease (CKD), and ischemic heart disease), chest X-ray (PA view), mechanical ventilator requirements, duration of hospitalization, laboratory parameters (hemoglobin, hematocrit, mean corpuscular volume (MCV), mean corpuscular hemoglobin (MCH), mean corpuscular hemoglobin concentration (MCHC), platelet counts, serum urea, serum creatinine, total leukocyte counts (including neutrophil and lymphocyte percentage), uric acid, serum electrolytes (Na, Cl, and K), alanine aminotransferase (ALT), aspartate aminotransferase (AST), alkaline phosphatase (ALP), total protein (albumin and globulin), and total, indirect, and direct bilirubin values), C-reactive protein (CPR), lactate dehydrogenase (LDH), low-density lipoproteins (LDL), ferritin, partial thromboplastin time (PT), activated partial thromboplastin time (APTT), interleukin-6 (IL6), cycle threshold value (CT value), serum D‑dimer, and disease outcomes (discharged or dead). The acquired data was checked and validated by the study’s researchers. Entries with incomplete information or dissonance were assessed by a distinct, professional quality assurance officer (Deputy Registrar), and adjustments were made where practicable.

Statistical analysis

Mortality and being discharged healthy were the main end results. Data were analyzed using several cross-tabulations to determine whether the variables are strongly associated with mortality. Categorical variables were recorded as percentages and frequencies. Continuous variables were recorded as means and standard deviations (SD). Data were checked for normality before statistical analysis. Normally distributed continuous variables were compared using the unpaired t‑test, whereas the Mann-Whitney U test was used for variables that were not normally distributed. Categorical variables were analyzed using either the chi‑square test or Fisher’s exact test (when more than 20% of cells have expected frequencies < 5). The level of significance was set at p<0.05. All analyses were done using SPSS version 18.0 (IBM Corp., Armonk, NY, USA).

Ethical consideration

All ethical issues were followed during the study. No identifying data was recorded. It was assured that all data collected was used only for the current study. The study was initiated after obtaining ethical approval from the Institutional Ethical and Review Board (IERB), AIIMS, Raipur (letter number OW/RC/AIIMS-RPR/2021/559, dated May 5, 2021).

## Results

Over 1,500 patients were admitted to the hospital until the first week of August 2021, but only 304 patient records were analyzed, and others were excluded due to missing or incomplete laboratory reports, missing X-rays, incomplete clinical notes, etc.

Table 1 shows that the mean age (in years) of patients was 53.77±15.85. Male patients were 27.3%, and females were 72.7%. The commonest comorbidities among the patients included hypertension (63.5%), followed by diabetes mellitus (34.2%), coronary artery disease (CAD) (23.4%), obesity (20.1%), chronic kidney disease (6.6%), TB (3.3%), hypothyroidism (2.6%), asthma/COPD (1.6%), and cancer (1.3%). The clinical signs and symptoms among the patients included most commonly fever/malaise (99.3%), followed by sore throat (98.4%), shortness of breath (94.1%), sough (93.8%), chest pain (77.6%), myalgia (77.3%), and anosmia/ageusia (75.7%). Of the patients, 84.2% have moderate to severe disease, and 15.8% have mild disease. Of the subjects, 26.3% were deceased, while 73.7% were discharged.

**Table 1:** Baseline and clinical characteristics of the study subjects (N=304).

Baseline and clinical characteristics	Number/mean	%/SD
Mean age (in years)	53.77	15.85
Gender		
Male	221	27.3
Female	83	72.7
Comorbidities		
Hypertension	193	63.5
Diabetes mellitus	104	34.2
Obesity	61	20.1
Asthma/COPD	5	1.6
TB	10	3.3
Chronic kidney disease	20	6.6
Cancer	4	1.3
Hypothyroidism	8	2.6
CAD	71	23.4
Signs and symptoms		
Fever/malaise	302	99.3
Shortness of breath	286	94.1
Chest pain	236	77.6
Cough	285	93.8
Sore throat	299	98.4
Anosmia/ageusia	230	75.7
Myalgia	235	77.3
Disease severity		
Moderate to severe	256	84.2
Mild	48	15.8
Disease outcome		
Discharge	224	73.7
Death	80	26.3

The laboratory parameter analysis reflected that total leukocyte counts (/mm^3^) were raised in patients with severe to moderate disease (12.69±6.98) as compared to patients with mild disease (9.22±5.15) with a p-value of <0.05. Also, serum urea (mg/dL) levels were significantly (p<0.05) raised in severe and moderate patients (78.04±62.44) compared to mild patients (33.09±22.79). Other laboratory parameters that were significantly (p<0.05) raised among severe and moderate compared to mild patients included CT value (positive if <35), total leukocyte counts (/mm^3^), neutrophils (%), absolute neutrophil count (/mm^3^), serum urea (mg/dL), serum uric acid (mg/dL), serum sodium (Na) (mEq/L), serum total bilirubin (mg/dL), serum indirect bilirubin (mg/dL), serum direct bilirubin (mg/dL), serum AST (IU/L), serum ALT (IU/L), serum alkaline phosphatase (IU/L), globulin (g/dL), CRP (mg/L), and PT (seconds) (Table 2).

**Table 2:** Comparison of hematological and biochemical characteristics with disease severity among the study subjects (N=304).

Biochemical characteristics	Disease severity (mean±SD)		p-value
	Severe/moderate (n=256)	Mild (n=48)	
CT value	16.58±6.13	3.52±1.47	<0.05
Hemoglobin (g/dL)	12.45±2.46	12.63±2.06	>0.05
Hematocrit (%)	38.43±7.35	39.07±5.62	>0.05
Total leukocyte counts (/mm^3^)	12.69±6.98	9.22±5.15	<0.05
Neutrophils (%)	80.09±13.90	63.50±16.73	<0.05
Lymphocytes (%)	12.02±10.36	25.61±13.81	<0.05
Absolute neutrophil count (/mm^3^)	10.81±7.01	5.48±4.16	<0.05
Absolute lymphocyte count (/mm^3^)	1.16±1.04	1.71±0.96	<0.05
Total platelet counts (lacs/mm^3^)	244.93±123.78	252.73±121.84	>0.05
MCV (fL)	86.51±10.32	85.35±11.25	>0.05
MCH (pg)	32.17±55.03	27.51±4.26	>0.05
MCHC (g/dL)	32.46±1.73	32.21±1.82	>0.05
Serum urea (mg/dL)	78.04±62.44	33.09±22.79	<0.05
Serum creatinine (mg/dL)	1.87±2.03	1.42±2.12	>0.05
Serum uric acid (mg/dL)	5.57±3.14	4.64±1.27	<0.05
Serum Na (mEq/L)	141.29±9.87	139.15±2.64	<0.05
Serum K (mEq/L)	4.51±1.07	4.20±0.52	>0.05
Serum Cl (mEq/L)	104.00±9.61	103.76±3.49	>0.05
Serum total bilirubin (mg/dL)	0.89±0.69	0.58±0.27	<0.05
Serum direct bilirubin (mg/dL)	0.31±0.41	0.16±0.12	<0.05
Serum indirect bilirubin (mg/dL)	0.57±0.33	0.43±0.17	<0.05
Serum AST (IU/L)	69.56±94.94	33.73±14.38	<0.05
Serum ALT (IU/L)	64.08±88.54	39.17±29.46	<0.05
Serum alkaline phosphatase (IU/L)	101.33±62.03	84.30±38.65	<0.05
Total protein (g/dL)	6.29±0.83	9.18±15.19	>0.05
Albumin (g/dL)	3.09±0.53	3.94±0.75	<0.05
Globulin (g/dL)	3.20±0.60	2.96±0.52	<0.05
CRP (mg/L)	88.11±63.87	27.31±39.93	<0.05
LDL (mg/dL)	93.08±78.93	77.01±33.08	>0.05
PT (in seconds)	11.95±2.08	10.54±0.57	<0.05
APTT (in seconds)	33.68±18.07	27.95±4.08	>0.05
IL6 (pg/mL)	154.16±446.08	64.04±76.76	>0.05
Calcium (mg/L)	8.65±0.51	8.81±1.20	>0.05
Phosphate (mg/L)	4.63±2.22	4.18±1.08	>0.05
HbA1c (%)	8.56±2.26	9.78±2.95	>0.05

In the present study, the biochemical parameter analysis reflected that total leukocyte counts (/mm^3^) were raised in deceased patients (15.03±7.55) as compared to discharged patients (11.11±6.26) with a p-value of <0.05. Also, serum urea (mg/dL) levels were significantly raised (p<0.05) in dead patients (99.53±71.64) compared to discharged patients (60.45±51.91). Other laboratory parameters that were significantly (p<0.05) raised among the deceased compared to discharged patients included hematocrit (%), total leukocyte counts (/mm^3^), neutrophils (%), MCH (pg), serum urea (mg/dL), serum uric acid (mg/dL), serum Na (mEq/L), serum Cl (mEq/L), serum total bilirubin (mg/dL), serum indirect bilirubin (mg/dL), serum ALT (IU/L), PT (seconds), and serum phosphate (mg/L) (Table 3).

**Table 3:** Comparison of hematological and biochemical characteristics with disease mortality among the study subjects (N=304).

Biochemical characteristics	Disease outcome (mean±SD)		p-value
	Discharge (n=224)	Dead (n=80)	
CT value	12.49±7.32	20.16±3.88	<0.05
Hemoglobin (g/dL)	12.44±2.22	12.59±2.84	>0.05
Hematocrit (%)	38.29±6.28	39.24±9.02	<0.05
Total leukocyte counts (/mm^3^)	11.11±6.26	15.03±7.55	<0.05
Neutrophils (%)	74.70±16.01	84.97±11.51	<0.05
Lymphocytes (%)	16.05±12.57	9.08±8.64	<0.05
Absolute neutrophil count (/mm^3^)	8.70±6.24	13.53±7.52	>0.05
Absolute lymphocyte count (/mm^3^)	1.31±0.99	1.06±1.18	>0.05
Total platelet counts (lacs/mm^3^)	255.33±121.25	220.52±124.25	>0.05
MCV (fL)	86.02±10.95	87.17±9.01	>0.05
MCH (pg)	28.34±5.23	40.01±97.84	<0.05
MCHC (g/dL)	32.48±1.68	32.24±1.90	>0.05
Serum urea (mg/dL)	60.45±51.91	99.53±71.64	<0.05
Serum creatinine (mg/dL)	1.69±1.82	2.09±2.57	>0.05
Serum uric acid (mg/dL)	5.17±2.62	6.12±3.62	<0.05
Serum Na (mEq/L)	140.32±7.89	142.70±11.85	<0.05
Serum K (mEq/L)	4.41±0.88	4.59±1.30	>0.05
Serum Cl (mEq/L)	103.43±7.60	105.42±11.77	<0.05
Serum total bilirubin (mg/dL)	0.76±0.48	1.08±0.95	<0.05
Serum direct bilirubin (mg/dL)	0.24±0.21	0.41±0.64	<0.05
Serum indirect bilirubin (mg/dL)	0.52±0.29	0.61±0.37	<0.05
Serum AST (IU/L)	57.02±77.83	82.61±110.31	>0.05
Serum ALT (IU/L)	54.25±65.85	76.20±115.67	<0.05
Serum alkaline phosphatase (IU/L)	95.15±62.13	108.39±49.74	>0.05
Total protein (g/dL)	6.96±7.12	6.16±0.86	>0.05
Albumin (g/dL)	3.34±0.66	2.91±0.47	<0.05
Globulin (g/dL)	3.13±0.57	3.25±0.65	>0.05
CRP (mg/L)	70.88±61.64	105.28±65.32	<0.05
LDL (mg/dL)	87.03±68.35	81.833±23.55	>0.05
PT (in seconds)	11.41±1.70	12.591±2.42	<0.05
APTT (in seconds)	32.54±18.70	33.13±9.15	>0.05
IL6 (pg/mL)	117.37±279.23	205.50±675.47	>0.05
Calcium (mg/L)	8.78±0.89	8.37±0.38	>0.05
Phosphate (mg/L)	4.35±1.33	5.33±5.43	<0.05
HbA1c (%)	8.52±2.15	9.42±2.98	>0.05

## Discussion

The COVID-19 epidemic is rapidly spreading over the world, putting a strain on healthcare systems. The unpredictable course of sickness, which can range from asymptomatic to severely ill with acute respiratory failure complications, necessitates gathering adequate data to accurately diagnose the patient’s progress and determine complications [11-13].

The proportion of COVID-19 cases in India is increasing, but mortality had continued to be lesser than those in other nations with comparable numbers of COVID-19 infections. The mortality rate was found to be 26.3% in this study, which is significantly higher than in developed nations [14-17]. Although India was thought to be under rigorous countrywide lockout, the results of a study found that the majority of Indians seemed to have supportive attitudes and intentions regarding shutdown regulations [18-20]. Following that, India experienced a disproportionately higher risk of death. Furthermore, subsequent relaxing of restrictions in India and increased movement have resulted in an increase in the disease and reported deaths [21,22].

In the present study, lymphocyte (%) was decreased in COVID-19 patients (12.02±10.36 in severe/moderate cases versus 25.61±13.81 in mild cases), and it was significantly associated with the severity of the disease (p<0.05). These findings were consistent with the studies conducted by Huang et al. [23] and Patel et al. [24]. According to one theory, lymphocytes that carry the SARS-CoV-2 receptor ACE2 are immediately targeted and destroyed by the virus [25]. Another suggestion seems to be that in COVID-19 cases, elevated pro-inflammatory cytokine levels such as tumor necrosis factor (TNF) and IL-6 promote the apoptosis of lymphocytes [26]. As a result, lymphopenia suppresses the body’s natural (innate) immunity, worsening COVID-19 symptoms and resulting in a poor prognosis.

CRP seems to be an acute-phase reactant (nonspecific) that is produced in the liver as a result of IL-6. Raised serum levels of CRP are linked to the severity of disease and degree of inflammation [27]. In the present study, CRP (mg/dL) levels were elevated with the increased disease severity (88.11±63.87 severe/moderate cases versus 27.31±39.93 in mild cases; p<0.05) and poor disease outcome (105.28±65.32 in deceased versus 70.88±61.64 in discharged; p<0.05), and it was in coherence with the studies done by Liu et al. [28] and Qin et al. [29].

It was found in the present study that elevated IL-6 levels were significantly associated with an increased risk of poor outcomes (205.50±675.47 in deceased versus 117.37±279.23 in discharged; p<0.05) among COVID-19 patients. COVID-19’s enhanced inflammatory response, combined with hypoxia from severe pneumonia, results in coagulation and fibrinolysis activation, leading to a hypercoagulable condition that causes disseminated intravascular coagulation (DIC) and multi-organ failure [30].

The present study found that liver function tests were significantly deranged with the increased severity of the disease (p<0.05). In a study done by Cai et al., 76.3% of COVID-19 patients had abnormal liver tests (ALT, AST, AP, and total bilirubin), and in COVID-19, ALT and AST levels are transiently increased, and the mechanism through which liver dysfunction occurs is most likely through secondary liver damage rather than a direct insult [28].

Creatinine levels were shown to rise with the severity of the disease (p<0.05) in the current study. The risk of acute renal injury and mortality following hospital admission was considerably greater in patients having raised baseline serum creatinine levels than in individuals having baseline values within the normal range, according to prospective cohort research done among COVID-19 patients (n=701). A reason for this has been thought to be blood transmission and retention of the virus in the renal system, resulting in renal cell necrosis [29].

Limitations

This research has a few limitations. First and foremost, this was a single-center study. Multicenter research with a larger sample size will aid in a better understanding of COVID-19 patients’ clinical and laboratory profiles and outcomes. Another limitation was that confounders were not addressed during the analysis.

## Conclusions

Clinical and biochemical characteristics reflect disease pathophysiology and hence aid doctors in determining the severity of diseases. It can help in the creation of algorithms for the management and care of patients to enhance disease outcomes. These traits will aid in the identification of critically severe patients and the deployment of care services. These attributes may also aid in the prevention of COVID-19-induced acute inflammatory response consequences such as respiratory arrest, shock, or multiple organ failure in patients.
